# Green Extraction Approach for Phenolic‐Rich Peppermint Extracts Using Ultrasound: Impact of Solvent and Time Variables

**DOI:** 10.1155/tswj/4620252

**Published:** 2026-09-12

**Authors:** Kantapon Premprayoon, Tossapol Jangnoi, Likit Temprom, Khakhanang Ratananikom

**Affiliations:** ^1^ Department of Agricultural Machinery Engineering, Faculty of Engineering, Rajamangala University of Technology Isan, Khon Kaen Campus, Khon Kaen, Thailand, rmuti.ac.th; ^2^ Department of Mechanical Engineering, Faculty of Engineering, Rajamangala University of Technology Isan, Khon Kaen Campus, Khon Kaen, Thailand, rmuti.ac.th; ^3^ Department of Applied Physics, Faculty of Engineering, Rajamangala University of Technology Isan, Khon Kaen Campus, Khon Kaen, Thailand, rmuti.ac.th; ^4^ Department of Public Health, Faculty of Science and Health Technology, Kalasin University, Kalasin, Thailand, ksu.ac.th

**Keywords:** antioxidant activity, green extraction, *Mentha piperita* L., phenolic compounds, ultrasound-assisted extraction

## Abstract

This study investigated the effects of solvent composition and sonication time on the ultrasound‐assisted extraction of phenolic‐rich peppermint extracts using ethanol–water mixtures. Ethanol concentrations of 0%, 50%, 75%, and 95% were applied for extraction periods of 15 and 30 min, and the resulting extracts were evaluated for total phenolic content (TPC), total flavonoid content (TFC), and antioxidant activities using DPPH and ABTS assays. Two‐way ANOVA revealed that ethanol concentration, sonication time, and their interaction significantly affected all measured parameters (*p* < 0.01). The optimal extraction condition was 50% ethanol for 15 min, which produced the highest TPC (141.64 ± 3.34 mg GAE/g extract) and TFC (256.20 ± 2.43 mg RE/g extract), together with the strongest antioxidant activities, as indicated by the lowest IC_50_ values for DPPH (0.123 ± 0.001 mg/mL) and ABTS (0.023 ± 0.001 mg/mL) radical scavenging activity. Correlation analysis demonstrated strong negative relationships between phytochemical contents and antioxidant IC_50_ values, indicating that increased phenolic and flavonoid contents were associated with enhanced antioxidant activity. Prolonged sonication generally reduced extraction efficiency under certain solvent conditions, particularly at intermediate ethanol concentrations, suggesting possible degradation of sensitive phytochemicals during extended cavitation. These findings demonstrate the importance of balancing solvent polarity and extraction duration to maximize phytochemical recovery and antioxidant activity under green extraction conditions.

## 1. Introduction

In recent years, the pursuit of sustainable, high‐efficiency extraction technologies for bioactive compounds from plant sources has intensified, driven by the growing demand for natural antioxidants in functional foods, nutraceuticals, and pharmaceuticals. Phytochemicals such as polyphenols and flavonoids are recognized for their antioxidant, anti‐inflammatory, antimicrobial, and anticancer activities, making them valuable for replacing synthetic additives in consumer products [1–4]. Consumer preference is increasingly shifting toward plant‐based and environmentally friendly solutions, creating both an industrial and scientific imperative to optimize extraction processes for maximum yield and stability of bioactive compounds [5, 6].

Conventional extraction techniques, including maceration, Soxhlet, and reflux, are widely used but often involve prolonged processing, high solvent volumes, and elevated temperatures, which can degrade thermolabile compounds [7, 8]. In contrast, ultrasound‐assisted extraction (UAE) has emerged as a green, scalable alternative. By generating acoustic cavitation, UAE disrupts plant cell walls and enhances solvent penetration, enabling rapid and efficient recovery of phytochemicals with lower solvent consumption and reduced processing times [9–11].

Peppermint (*Mentha piperita* L.), a natural hybrid of watermint (*M. aquatica*) and spearmint (*M. spicata*), is widely used in food, beverage, and pharmaceutical industries for its distinctive aroma and bioactive profile, including phenolic acids (e.g., rosmarinic acid and caffeic acid), flavonoids (e.g., eriocitrin and luteolin), and volatile terpenoids (e.g., menthol and menthone). These compounds contribute to its potent antioxidant, antimicrobial, and anti‐inflammatory activities, making peppermint a valuable candidate for functional food and nutraceutical applications. [12–15]. Although peppermint essential oils have been extensively characterized, fewer studies have systematically optimized UAE condition, particularly solvent polarity and extraction time, using food‐grade solvents suitable for functional food applications [16, 17], but these often use nonfood‐grade solvents, lack factorial optimization, or omit key antioxidant metrics, leaving an important gap in both applied and green extraction research.

This study addresses that gap by systematically optimizing ethanol concentration (0%–95%, v/v) and sonication time (15 vs. 30 min) for the UAE of total phenolic content (TPC), total flavonoid content (TFC), and antioxidant activity (DPPH and ABTS assays) from peppermint leaves. This work is distinctive in its exclusive use of food‐grade solvents, comparative evaluation of extraction parameters, and direct alignment with green chemistry principles. The findings provide actionable process parameters for developing phenolic‐rich peppermint extracts with strong antioxidant capacity, offering both scientific novelty and industrial relevance.

## 2. Material and Methods

### 2.1. Plant Material Collection and Preparation

Fresh peppermint (*M. piperita* L.) leaves were purchased from a local grower at a local market in Kalasin province, Thailand, in July 2025. The material was sold by the grower as peppermint. No manufacturer, batch/lot number, certificate of authentication, or herbarium voucher specimen were available because the material was obtained directly from a local grower rather than from a packaged commercial supplier. The plant material was identified based on the seller′s description and morphological characteristics of the dried leaves. Therefore, the absence of formal taxonomic authentication and batch‐level traceability is acknowledged as a limitation of the study.

### 2.2. UAE Procedure

UAE was conducted based on the method of Ratananikom and Premprayoon [18], with slight modifications. The extraction process was performed using an ultrasonic water bath (LabTech, Korea) operating at 50 kHz frequency and 350 W power. The independent variables included ethanol concentrations (0%, 50%, 75%, and 95% v/v) and extraction durations (15 and 30 min). A fixed sample‐to‐solvent ratio of 1:10 (w/v) was used across all treatments.

After sonication, the mixtures were centrifuged at 12,000 rpm for 10 min to separate solid residues. The resulting supernatants were collected and concentrated using a rotary evaporator. The crude extracts were stored at 4°C in amber vials until further analysis.

### 2.3. Determination of TPC

The TPC of peppermint extracts was determined using the Folin–Ciocalteu colorimetric assay as described by Singleton et al. [19], with minor modifications. In brief, 20 *μ*L of the extract was mixed with 500 *μ*L of 10% (v/v) Folin–Ciocalteu reagent and incubated at room temperature for 10 min. Subsequently, 1 mL of 7.5% (w/v) sodium carbonate solution was added, and the mixture was allowed to react for 60 min at room temperature. The absorbance was measured at 765 nm. The TPC was calculated from a gallic acid calibration curve and expressed as milligrams of gallic acid equivalents per gram of extract (mg GAE/g extract).

### 2.4. Determination of TFC

TFC was measured using the aluminum chloride colorimetric method, adapted from Wolfe et al. [20]. A 200 *μ*L of the extract was mixed with 150 *μ*L of 5% (w/v) sodium nitrite and allowed to stand for 5 min at room temperature. Next, 150 *μ*L of 10% (w/v) aluminum chloride was added and incubated for 6 min. Then, 500 *μ*L of 1 M sodium hydroxide was added, and the final volume was adjusted to 1.5 mL by adding 500 *μ*L of distilled water. The absorbance was recorded at 510 nm. The TFC was calculated using a rutin standard curve and expressed as milligrams of rutin equivalents per gram of extract (mg RE/g extract).

### 2.5. Determination of Antioxidant Activities

#### 2.5.1. DPPH Radical Scavenging Assay

The free radical scavenging activity of peppermint extracts was evaluated using the DPPH (2,2‐diphenyl‐1‐picrylhydrazyl) assay, following the method of Brand‐Williams et al. [21], with slight modifications. A 5 *μ*L of the extract was added to 1.5 mL of 0.1 mM DPPH solution in ethanol. The mixture was incubated in the dark at room temperature for 10 min, and the absorbance was measured at 517 nm. Scavenging activity was calculated using the equation:

DPPH scavenging activity %=Abscontrol−AbssampleAbscontrol×100,

where Abs_control_ is the absorbance of the control (DPPH solution without the extract) and Abs_sample_ is the absorbance in the presence of the extract. The antioxidant activity was reported as the half‐maximal inhibitory concentration (IC_50_).

#### 2.5.2. ABTS Radical Scavenging Assay

The ABTS [2,2 ^′^‐azino‐bis(3‐ethylbenzothiazoline‐6‐sulfonic acid)] assay was performed following the protocol of Re et al. [22], with slight modifications. The ABTS radical cation (ABTS^+^) was generated by mixing equal volumes of 7 mM ABTS solution and 2.45 mM potassium persulfate, then allowing the reaction to proceed in the dark at room temperature for 16 h. The working solution was diluted with distilled water to obtain an absorbance of 0.80 ± 0.02 at 734 nm.

A 5 *μ*L of the extract was added to 1.5 mL of the ABTS^+^ solution, incubated for 10 min at room temperature in the dark, and the absorbance was measured at 734 nm. The percentage of scavenging activity was calculated using the following equation:

ABTS scavenging activity %=Abscontrol−AbssampleAbscontrol×100,

where Abs_control_ is the absorbance of the control ABTS^+^ solution without the extract and Abs_sample_ is the absorbance after mixing with the extract. The antioxidant activity was reported as the half‐maximal inhibitory concentration (IC_50_).

### 2.6. Statistical Analysis

All experimental results were expressed as mean ± standard deviation (SD) from triplicate measurements. Data were analyzed using two‐way analysis of variance (two‐way ANOVA), followed by post hoc comparisons using the least significant difference (LSD) test to evaluate the effects of ethanol concentration, sonication time, and their interaction on phytochemical contents and antioxidant activities. All statistical analyses were carried out using Statistix software. Differences were considered statistically significant at *p* < 0.05.

Pearson′s correlation analysis was additionally performed to evaluate the linear relationships among TPC, TFC, and antioxidant activities determined by DPPH and ABTS radical scavenging assays. Correlation coefficients (*r*) and corresponding *p* values were calculated to assess the strength, direction, and statistical significance of the associations among variables.

## 3. Results

### 3.1. TPC and TFC of Peppermint Extracts

Table 1 shows the TPC and TFC of peppermint extracts. Both main effects (ethanol concentration and sonication time) and their interaction significantly affected TPC and TFC (*p* < 0.01). Two‐way ANOVA followed by LSD post hoc analysis revealed that 50% ethanol for 15 min produced the highest TPC (141.64 ± 3.34 mg GAE/g extract) and TFC (256.20 ± 2.43 mg RE/g extract), both of which were significantly higher than all other treatments (*p* < 0.05). In contrast, extraction with 0% ethanol (water) or 95% ethanol resulted in the lowest yields. Prolonged extraction to 30 min significantly reduced both TPC and TFC across most solvent systems (*p* < 0.05), suggesting degradation of phenolic and flavonoid compounds.

**Table 1:** Total phenolic content and total flavonoid content of peppermint extracts.

Ethanol (%)	Total phenolic content (mg GAE/g extract)		Total flavonoid content (mg RE/g extract)	
	Time (min)		Time (min)	
	15	30	15	30
0	45.27 ± 0.64^h^	55.64 ± 1.41^g^	56.80 ± 0.37^g^	70.33 ± 0.97^f^
50	141.64 ± 3.34^a^	110.95 ± 2.76^d^	256.20 ± 2.43^a^	243.96 ± 1.07^c^
75	130.00 ± 3.47^b^	113.86 ± 1.48^c^	249.73 ± 2.47^b^	242.18 ± 0.80^c^
95	68.91 ± 0.13^f^	76.82 ± 1.67^e^	204.27 ± 4.04^d^	181.58 ± 3.76^e^
F‐test	C ^∗∗^, T ^∗∗^, CxT ^∗∗^		C ^∗∗^, T ^∗∗^, CxT ^∗∗^	

*Note:* Values are expressed as mean ± SD. Mean values with different superscript letters are significantly different at *p* < 0.05 according to two‐way ANOVA followed by LSD test. C means ethanol concentration; T means sonication time; and CxT means interaction between ethanol concentration and sonication time.

^∗∗^Significant difference at *p* < 0.01.

In addition, a significant interaction between ethanol concentration and sonication time (C × T, *p* < 0.01) was observed for both TPC and TFC, indicating that the influence of sonication time varied according to ethanol concentration. At 50% and 75% ethanol concentrations, extending sonication time from 15 to 30 min significantly decreased the recovery of phenolic and flavonoid compounds, whereas extraction with water showed increased yields with prolonged sonication. These results demonstrated that solvent polarity and extraction duration jointly influenced the extraction efficiency of bioactive compounds from peppermint leaves.

### 3.2. Antioxidant Activities

#### 3.2.1. DPPH Radical Scavenging Activity

**Figure 1:**
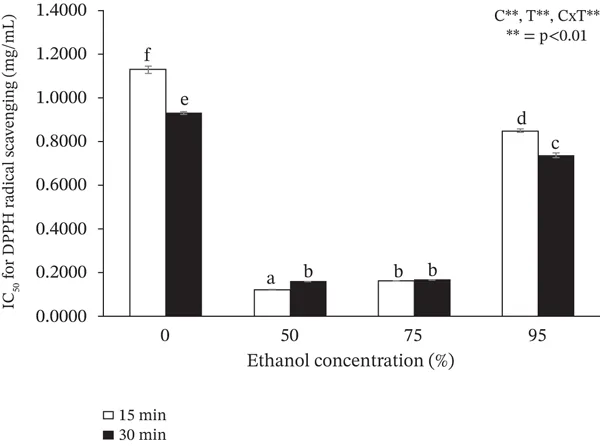
DPPH radical scavenging activity of peppermint extracts. *Note:* Bar chart with different superscript letters indicate significant difference at *p* < 0.05 according to two‐way ANOVA followed by LSD test. C means ethanol concentration. T means sonication time. CxT means interaction between ethanol concentration and sonication time.  ^∗∗^ means significant difference at *p* < 0.01.

Extraction conditions significantly affected the IC_50_ values of DPPH radical scavenging activity. The main effects of solvent concentration and extraction time, as well as their interaction, were statistically significant (*p* < 0.01). The lowest IC_50_, indicating the highest antioxidant activity, was obtained with 50% ethanol for 15 min (0.123 ± 0.001 mg/mL), which was significantly different from all other treatments (*p* < 0.05) (Figure 1).

In addition, a significant interaction between ethanol concentration and sonication time (C × T, *p* < 0.01) was observed, indicating that the effect of sonication time on antioxidant activity varied depending on solvent polarity. At 50% ethanol concentrations, prolonging sonication time resulted in increased IC_50_ value and reduced radical scavenging activity, whereas extraction with water (0% ethanol) and 95% ethanol showed a decrease in IC_50_ after longer sonication. These results suggested that solvent polarity and extraction duration jointly influenced the recovery and stability of antioxidant compounds in peppermint extracts.

#### 3.2.2. ABTS Radical Scavenging Activity

**Figure 2:**
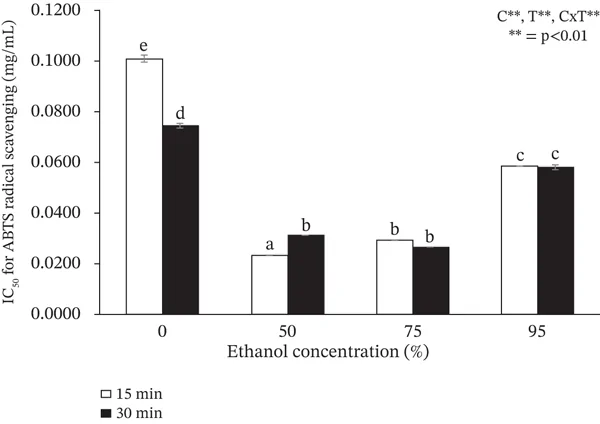
ABTS radical scavenging activity of peppermint extracts. *Note:* Bar chart with different superscript letters indicate significant difference at *p* < 0.05 according to two‐way ANOVA followed by LSD test. C means ethanol concentration. T means sonication time. CxT means interaction between ethanol concentration and sonication time.  ^∗∗^ means significant difference at *p* < 0.01.

A similar pattern was observed for ABTS radical scavenging activity. The main effects of solvent concentration and extraction time, as well as their interaction, significantly influenced IC_50_ values of ABTS radical scavenging activity (*p* < 0.01). The lowest IC_50_ value, indicating the strongest antioxidant activity, was obtained using 50% ethanol for 15 min (0.023 ± 0.001 mg/mL) (Figure 2).

Furthermore, a significant interaction between ethanol concentration and sonication time (C × T, *p* < 0.01) was observed for ABTS radical scavenging activity, indicating that the influence of sonication time varied according to solvent polarity. At 50% ethanol, prolonging sonication from 15 to 30 min significantly increased IC_50_ values, reflecting reduced antioxidant efficiency. In contrast, extracts obtained with 75% ethanol showed a slight decrease in IC_50_ after prolonged sonication, whereas the 95% ethanol treatment remained relatively unchanged. These findings demonstrated that solvent polarity and extraction duration collectively affected the antioxidant properties of peppermint extracts.

The consistency between DPPH and ABTS results indicated that both antioxidant assays responded similarly to extraction conditions and phytochemical contents of peppermint extracts.

### 3.3. Correlation Analysis

Table 2 presents the Pearson correlation coefficients among TPC, TFC, and antioxidant activities (the IC_50_ values of DPPH and ABTS radical scavenging activity). All variable comparisons had significant correlations (*p* < 0.01). The TPC showed a very strong positive correlation with TFC (*r* = 0.899, *p* < 0.01). In contrast, TPC exhibited very strong negative correlations with the IC_50_ value of DPPH (*r* = −0.970, *p* < 0.01) and ABTS (*r* = −0.954, *p* < 0.001) radical scavenging activity. Similarly, TFC was strongly negatively correlated with the IC_50_ value of DPPH (*r* = −0.905, *p* < 0.01) and ABTS (*r* = −0.936, *p* < 0.01) radical scavenging activity. A very strong positive correlation was also observed between the IC_50_ value of DPPH and ABTS radical scavenging activity (*r* = 0.968, *p* < 0.01).

**Table 2:** Pearson correlation coefficients among phytochemical contents and antioxidant activities.

Variables	TPC	TFC	DPPH	ABTS
TPC	1.000	0.899 ^∗∗^	−0.970 ^∗∗^	−0.954 ^∗∗^
TFC	0.899 ^∗∗^	1.000	−0.905 ^∗∗^	−0.936 ^∗∗^
DPPH	−0.970 ^∗∗^	−0.905 ^∗∗^	1.000	0.968 ^∗∗^
ABTS	−0.954 ^∗∗^	−0.936 ^∗∗^	0.968 ^∗∗^	1.000

*Note:* Values represent Pearson correlation coefficients (*r*).

^∗∗^Statistical significance at *p* < 0.01.

## 4. Discussion

The present study confirmed that ethanol concentration and UAE duration were critical factors in maximizing phenolic and flavonoid yields, as well as antioxidant capacity, from peppermint. Among the tested conditions, 50% ethanol for 15 min achieved the highest TPC (141.64 ± 3.34 mg GAE/g extract) and TFC (256.20 ± 2.43 mg RE/g extract), coupled with the strongest radical scavenging activity in both DPPH (IC_50_ = 0.123 ± 0.001 mg/mL) and ABTS (IC_50_ = 0.023 ± 0.001 mg/mL) assays.

Compared with the maceration‐based extraction of peppermint reported by Farnad et al. [23], which yielded substantially lower TPC and TFC values, the UAE approach used in the present study produced markedly higher phytochemical recovery within a considerably shorter extraction time, highlighting the efficiency of UAE. The improved TPC and TFC observed following UAE treatment may be attributed to ultrasonic cavitation, which disrupts plant cell structures and weakens cell wall integrity, thereby enhancing solvent penetration and facilitating the release of phenolic and flavonoid compounds.

Similar improvements in phytochemical recovery and antioxidant activity following UAE treatment have been reported in various herbal materials. For dill (*Anethum graveolens* L.), extraction with 50% ethanol for 30 min produced the highest TPC (135.88 ± 3.23 mg GAE/g extract) and TFC (229.53 ± 4.97 mg RE/g extract), together with strong antioxidant activities indicated by low IC_50_ values against DPPH (0.12 ± 0.00 mg/mL) and ABTS (0.034 ± 0.00 mg/mL) radicals [18]. Nevertheless, the effectiveness of UAE may vary depending on the characteristics of the plant matrix. Ramli et al. [24] reported that UAE enhanced flavonoid recovery and antioxidant activity in red dragon fruit peel while reducing extraction yield, whereas the opposite trend was observed in the fruit flesh. These findings suggested that the response to UAE is strongly influenced by the physicochemical properties of plant materials. Therefore, extraction parameters, particularly solvent composition and sonication duration, should be individually optimized to maximize phytochemical recovery and antioxidant performance.

The present study demonstrated that intermediate ethanol concentrations produced higher phenolic recovery and antioxidant activity than absolute ethanol or water alone. This observation may be attributed to the balanced polarity of ethanol–water mixtures, which facilitates the coextraction of both hydrophilic and moderately lipophilic phenolic compounds. In contrast, excessively polar or nonpolar solvents may limit the solubilization of diverse phenolic constituents. Similar trends have been reported in previous studies on polyphenol‐rich herbs, where ethanol–water mixtures enhanced phytochemical extraction more effectively than single‐solvent systems [9, 18, 25–27].

The effect of sonication duration on phytochemical recovery depended on solvent concentration, with the most pronounced reduction in TPC, TFC, and antioxidant activity observed in extracts prepared with 50% ethanol after extending sonication time from 15 to 30 min in the present study. Prolonged sonication generally reduced yields and antioxidant capacity, suggesting oxidative or thermal degradation of sensitive phytochemicals under extended cavitation [28–32]. Therefore, our findings indicated that extraction efficiency depends on both the balance between solvent polarity and sonication duration, with moderate ethanol concentration and short extraction time providing favorable conditions for maximizing phytochemical recovery while minimizing antioxidant degradation. In addition, the significant interaction effects identified through two‐way ANOVA and the strong correlations between phytochemical contents and antioxidant activities provided further evidence supporting the optimized extraction conditions proposed in this study.

However, the present study did not include direct chromatographic identification or quantification of individual bioactive compounds. The discussion of specific phytochemicals was therefore based on previously reported compounds in peppermint extracts from the literature. The previous researchers have identified several individual bioactive compounds in peppermint that contribute to its antioxidant capacity. The major phenolic acid reported in peppermint leaves is rosmarinic acid, a caffeic acid derivative known for its strong radical‐scavenging and metal‐chelating activities [15, 33]. Flavonoids such as eriocitrin, luteolin, and hesperidin have also been documented, with eriocitrin being particularly abundant in peppermint and recognized for its potent antioxidant and anti‐inflammatory properties [12–15, 34, 35]. Other compounds, including chlorogenic acid and apigenin derivatives, have been detected in ethanol–water extracts, further contributing to their biological activity. Given the polarity range of these compounds, the observed superiority of 50% ethanol for 15 min in our study is consistent with literature reports, as this solvent ratio efficiently solubilizes both moderately polar phenolic acids and less polar flavonoids. Future work incorporating high‐performance liquid chromatography (HPLC) or liquid chromatography–mass spectrometry (LC‐MS) could provide a detailed phytochemical profile to directly link compound‐level composition with the antioxidant activities observed here.

In addition, another limitation of this study is that the peppermint material was obtained from a local grower at a local market, and no herbarium voucher specimen, certificate from an authorized grower, manufacturer information, or batch/lot number were available. Therefore, although the material was sold as peppermint and was assessed based on morphological characteristics, complete taxonomic authentication and batch‐level traceability could not be confirmed. Future studies should use botanically authenticated plant material deposited in an official herbarium or material accompanied by a certificate of authentication to improve reproducibility.

The correlation analysis revealed very strong positive relationships between TPC and TFC (*r* = 0.899, *p* < 0.01), indicating that extracts with higher phenolic contents also tended to contain higher flavonoid levels. In contrast, TPC exhibited very strong negative correlations with the IC_50_ value of DPPH (*r* = −0.970, *p* < 0.01) and the IC_50_ value of ABTS (*r* = −0.954, *p* < 0.01) radical scavenging activity, whereas TFC also showed strong negative correlations with IC_50_ value of DPPH (*r* = −0.905, *p* < 0.01) and ABTS radical scavenging activity (*r* = −0.936, *p* < 0.01). These findings indicated that increased phenolic and flavonoid contents were associated with stronger antioxidant activity. This observation is consistent with previous reports, highlighting the important role of phenolic compounds as hydrogen atom donors and electron transfer agents contributing to radical scavenging activity [36–39].

The slightly stronger correlations observed between TPC and antioxidant activities compared with TFC suggested that nonflavonoid phenolic compounds may also substantially contribute to the antioxidant properties of peppermint extracts. Previous studies have similarly reported that phenolic acids, including rosmarinic acid and caffeic acid derivatives, play important roles in the antioxidant activity of peppermint and related herbs. In addition, the very strong positive correlation between the IC_50_ value of DPPH and ABTS radical scavenging activity (*r* = 0.968, *p* < 0.01) indicated that both assays responded consistently to variations in phytochemical composition, despite differences in radical species and reaction mechanisms. These results reinforced the importance of employing multiple antioxidant assays to achieve a more comprehensive evaluation of antioxidant capacity [36–39].

The present study provides a practical evaluation of solvent concentration and short ultrasound extraction time on the recovery of phenolic‐rich peppermint extracts. Although the approach is not entirely novel, the findings contribute useful comparative data on the interaction between ethanol concentration and sonication duration, supported by two‐way ANOVA and correlation analysis between phytochemical contents and antioxidant activities.

The optimized UAE condition identified in this study (50% ethanol, 15 min) offers strong potential for industrial‐scale production of antioxidant‐rich peppermint extracts. This process is compatible with food‐grade manufacturing standards, as ethanol–water mixtures are safe, renewable, and widely accepted in the food and nutraceutical industries. Short extraction times reduce energy consumption, whereas ethanol can be efficiently recovered and reused, lowering operational costs and environmental impact. The resulting extracts can be incorporated into functional beverages, herbal teas, dietary supplements, or encapsulated powder formulations, offering both antioxidant benefits and clean‐label appeal to consumers. Furthermore, the mild processing conditions help preserve heat‐sensitive phytochemicals, potentially improving product stability and shelf life. Future research should explore pilot‐scale trials, solvent recovery efficiency, and integration into continuous extraction systems to fully validate commercial feasibility.

## 5. Conclusion

This study demonstrated that solvent polarity and sonication duration significantly influenced the UAE of phenolic and flavonoid compounds from peppermint. Among the investigated conditions, extraction using 50% ethanol for 15 min produced the highest total phenolic and flavonoid contents together with the strongest antioxidant activities. The results further indicated that extraction efficiency depended on the balance between solvent composition and extraction time, as prolonged sonication under certain solvent conditions reduced phytochemical recovery and antioxidant capacity. Correlation analysis confirmed strong relationships between phytochemical contents and antioxidant activities, highlighting the important contribution of phenolic compounds and flavonoids to the radical scavenging properties of peppermint extracts. Overall, the findings support the potential application of short‐duration UAE using food‐grade ethanol–water mixtures as an environmentally friendly approach for obtaining antioxidant‐rich peppermint extracts.

## Author Contributions

Conceptualization: K.R.; methodology: K.R.; investigation: K.R. and K.P.; formal analysis: K.R., K.P., T.J., and L.T.; data curation: K.R., K.P., T.J., and L.T.; writing—original draft preparation: K.R.; writing—review and editing: K.R., K.P., T.J., and L.T.; visualization: K.R.; supervision: K.R. and K.P.; project administration: K.R. and K.P.

## Funding

This research was financially supported by Thailand Science Research and Innovation via Rajamangala University of Technology Isan, Khon Kaen Campus (Grant Number: FF68/KKC/043).

## Disclosure

All authors have read and agreed to the published version of the manuscript.

## Conflicts of Interest

The authors declare no conflicts of interest.

## Data Availability

The data that support the findings of this study are available from the corresponding author upon reasonable request.
